# Effects of Hearing Loss on Semantic Prediction: Delayed Prediction for Intelligible Speech When Listening Is Demanding

**DOI:** 10.1097/AUD.0000000000001679

**Published:** 2025-06-19

**Authors:** Leigh B. Fernandez, Muzna Shehzad, Lauren V. Hadley

**Affiliations:** 1 Department of Social Sciences, Psycholinguistics Group, RPTU Kaiserslautern-Landau, Kaiserslautern, Germany; 2 Hearing Sciences - Scottish Section, School of Medicine, University of Nottingham, Glasgow, United Kingdom.

**Keywords:** Semantic prediction, Age-related hearing loss, Listening demand, Visual world paradigm

## Abstract

**Objectives:**

Linguistic context can be used during speech listening to predict what a talker will say next. These predictions may be particularly useful in adverse listening conditions, since they can facilitate speech processing. In this study, we investigated the impact of postlingual hearing loss on prediction processes. Because hearing loss leads to a perceptual deficit (i.e., degraded auditory input), that can also have cognitive impacts (i.e., increased competition for cognitive resources due to increased listening effort), it is a naturalistic test case of how different sorts of challenge affect prediction.

**Design:**

We report a visual world eye-tracking study run with 3 participant groups: older adults (range: 53 to 80 years old) with normal hearing (n = 30), older adults with hearing loss listening under low demand (n = 32), and older adults with hearing loss listening under high demand (n = 31). Using highly semantically constraining predictable sentences, we analyzed the timecourse of simple associative predictions based on the agent of the sentence (sub-experiment 1), and the timecourse by which these predictions were narrowed with additional constraint provided by the verb (sub-experiment 2).

**Results:**

Although there was no effect of group on early agent-based predictions, we saw that the buildup and tailoring of verb-based prediction was delayed with hearing loss and exacerbated by listening demand. As there was no comparable group difference for semantically unconstraining neutral sentences, this cannot be explained as a result of delayed lexical access in the hearing loss groups. We also assessed the cost of incorrect predictions but did not see any group differences.

**Conclusion:**

These findings indicate two separable stages of prediction that are differently affected by hearing loss and listening demand (potentially due to changes in listening effort), and reveal delayed prediction as a cognitive impact of hearing loss that could compound simple audibility effects.

## INTRODUCTION

Words in context are easier to process than words in isolation (Obleser 2014). A linguistic context, or frame generated by semantics, syntax, and prosody (among other cues), allows listeners to use prior information to generate expectations of what is likely to come next. Such predictions can facilitate speech processing in a number of ways: increasing accuracy by reducing the stimulus information required to identify words, increasing processing speed since predicted words can be identified and accessed more quickly, and increasing ease since the cognitive cost of comprehending (more) predicted words is reduced (Holt et al. 2021). These benefits may be particularly helpful for listeners experiencing difficulty, such as people with hearing loss (PwHL). In this study, we investigated the impact of postlingual hearing loss on the generation and timecourse of prediction. Because hearing loss leads to a perceptual deficit (i.e., degraded auditory input), that can also have cognitive impacts (i.e., increased competition for cognitive resources due to increased listening effort), it provides a naturalistic test case of how different sorts of challenge affect prediction. Furthermore, a more comprehensive understanding of the changes in speech processing that result from hearing loss could lead to practical applications in the clinic or in technology.

### Prediction During Speech Listening

While prediction can occur at a range of levels (e.g., conceptual, semantic, syntactic, and phonological), we focus here on semantic prediction: that is, the use of linguistic context to engage in the preactivation of semantic information before it is encountered. Because prediction necessitates preactivation of specific targets, its measurement must enable assessment of processing before the predicted word being heard. Prediction has therefore been addressed through two main literatures: one focusing on event-related potentials and one focusing on eye movement. In the former, N400 amplitude has been commonly used as an index of the ease of semantic access (being inversely related to how predictable a word is, e.g., Kutas & Hillyard 1980; DeLong et al. 2005). N400 amplitude is also greater for adverbs that increase the predictability of the following targets, indicating that it is not only an early response to predictions being fulfilled (Freunberger & Roehm 2017). Other neural indications of prediction that occur before the predicted word have also been reported, with a semantic readiness potential (that specifically relates to the meaning of the predicted word) being shown to occur before target word occurrence in highly constraining sentences (Grisoni et al. 2017).

In the eye-tracking literature, on the other hand, a specific paradigm has been critical in allowing assessment of prediction: the visual world paradigm (VWP), which we use in this study. In the VWP (Tanenhaus et al. 1995), participants view an array of objects while hearing a sentence relating to some (or all) of the objects in the array, and the timing of looks to different objects are analyzed. Given that there is a tight coupling between shifts of attention and linguistic processing, the timing of looks to particular objects can provide insight into predictive behavior (i.e., a target is considered to have been predicted if participants preferentially look to its image before it is heard). In their seminal study using the VWP, Altmann and Kamide (1999) found that people predictively look to the only edible object in a visual array after hearing *The boy will eat*… relative to *The boy will move…*, suggesting that listeners use verb semantic information to predict the only feasible (i.e., edible) object in the array. Since then, this type of semantic prediction has been replicated in different languages and with different groups of language users (Kamide et al. 2003; Boland 2005; Brouwer et al. 2019; Holt et al. 2021; Theimann et al. 2021; Garrido Rodrigues et al. 2023; Prystauka et al. 2024). The VWP has also been used to assess different stages of semantic prediction, including distinguishing relatively cost-free automatic associative predictions from more costly and nonautomatic predictions that require tailoring to additional context (Pickering & Gambi 2018; Corps et al. 2022), demonstrating its sensitivity to nuances of predictive processes. For example, using the VWP, Corps et al. (2022) found that listeners made early predictions to semantically related objects and were later to make predictions that required using real-world knowledge to narrow down predictions to a contextually appropriate target.

Importantly, however, the vast majority of work investigating prediction across both literatures has been conducted with young participants. Thus, while evidence for prediction in young adults is relatively clear (for reviews, see Kamide 2008; Huettig & Janse 2015; Staub 2015; Huettig & Mani 2016; Ferreira & Chantavarin 2018), the use of prediction across the lifespan remains contentious. Studies of older adults variously report increased prediction in comparison to younger adults (Huettig & Janse 2016; Baltaretu & Chambers 2018; Milburn et al. 2023), similar predictions made in a different way (Stine-Morrow et al. 1996, 2001, 2008; Miller et al. 2004, 2006; Rayner et al. 2006, 2011), or reduced prediction (Morrow et al. 1992; Dagerman et al. 2006; Wlotko et al. 2010, 2012; DeLong et al. 2012). Indeed, Payne and Silcox (2019, p. 217) summarize that studies “…have produced evidence for nearly every possible outcome of age-related change.” We propose that the uncontrolled presence of (age-related) hearing loss in older participants may contribute to these inconsistencies, as long-term changes in hearing may alter speech processing mechanisms, and related increases in listening difficulty may further confound findings.

### Prediction in Challenging Conditions

Although prediction has been robustly demonstrated in ideal listening conditions, several pockets of work investigating listening in adverse conditions have found prediction to be reduced or delayed. Such findings are particularly relevant given our focus on effects of hearing loss. In a comprehensive review of speech recognition in adverse listening conditions, Mattys et al. (2013) differentiate issues due to (i) a degraded speech signal (e.g., due to background noise or a strong accent), and (ii) a limitation of the listener (e.g., due to increased cognitive load). We consider how prediction may be affected by such adverse listening conditions in the following paragraphs (see also Fernandez et al. 2024b).

When the signal is poor, it is plausible that prediction would be relied upon to a greater degree to compensate for less reliable bottom-up processing (Pickering & Garrod 2007; Van Os et al. 2022). For example, if the acoustic signal is masked with noise, listeners may use top-down context-driven predictions to aid in processing of degraded incoming information (Craik 2007; Pichora-Fuller 2009). Indeed, for people with normal hearing (PwNH), word recognition in noise is better when the target word is embedded in a predictable relative to a less predictable context (Kalikow et al. 1977; Boothroyd & Nittrouer 1988; Dubno et al. 2000). However, research has found that relying on top-down predictions can contradictorily lead to increased rates of mishearing in noise, particularly for older adults (Rogers et al. 2012; Sommers et al. 2015; Failes et al. 2020; Failes & Sommers 2022), and that noise-induced misunderstandings early on in a sentence can lead to inaccurate predictions that progressively impair word recognition as the sentence unfolds (Marrufo-Pérez et al. 2020). Hence while prediction may be useful in degraded listening situations, the costs of inaccurate prediction may outweigh the benefits.

The cognitive cost of generating the predictions in the first place may also impact their use in adverse conditions, as prediction has been suggested to require executive resources such as working memory (Huettig & Janse 2016) and inhibitory control (Cahana-Amitay et al. 2016). Previous research has found reduced linguistic prediction in groups of speakers with fewer available executive resources (Huettig & Mani 2016; Pickering & Gambi 2018) such as children (Borovsky et al. 2012; Gambi et al. 2018), older adults (Huettig & Mani 2016; Harel-Arbeli et al. 2023), and second language speakers (Lew-Williams & Fernald 2010; Grüter et al. 2012; Ito et al. 2017)*. Using the VWP, Huettig and Janse (2016) investigated the importance of a range of individual differences on anticipatory eye movements in participants ranging in age from 32 to 77 years, and found working memory and processing speed to independently contribute the most variance in anticipatory eye movements. Thus, a variety of studies indicates that if the speech signal is poor, or the listener’s cognitive resources are taxed, the ability to efficiently make predictions may be reduced.

### Perceptual and Cognitive Impacts of Hearing Loss

Hearing loss affects speech processing in a range of different ways. As well as affecting sound discrimination and speech recognition, hearing loss can increase listening demands (i.e., the complexity of the listening task), and has thus been proposed to require greater cognitive resources or effort allocation for speech processing (Pichora-Fuller et al. 2016; Peelle 2018). Indeed increases in listening effort with hearing loss have been demonstrated even for highly intelligible speech (Zekveld et al. 2011; but see (Ohlenforst et al. 2017) for a systematic review finding only weak evidence for a link between hearing loss and effort). Thus increased demands could compound perceptual difficulties, and have been related to increased errors during listening (Rabbitt 1968, 1990), difficulty with complex syntax (Wingfield et al. 2006; DeCaro et al. 2016), and reduced memory for linguistic information (Rabbitt 1968, 1990; Piquado et al. 2010). Finally, a higher incidence of mishearing with hearing loss (either due to a poorly perceived signal, higher listening demands, or fewer available cognitive resources) could further impact speech processing because this may lead to inference errors or the need to retrospectively correct misunderstanding (Rogers et al. 2012; Sommers et al. 2015; Failes et al. 2020; Failes & Sommers 2022). Thus, hearing loss involves the processing of degraded auditory input, often leads to increased listening demands (which may reduce the availability of cognitive resources and increase effort), and may increase mishearings, each of which could affect predictive processing during speech listening.

### Hearing Loss and Prediction: Motivation for the Present Study

In this article, we explore the effects of age-related hearing loss and listening demands, using the terms “people with hearing loss” (PwHL) in contrast with “people with normal hearing” (PwNH). Research investigating context use has found that PwHL may benefit more from constraining context relative to PwNH (Pichora-Fuller et al. 1995; Gordon-Salant & Fitzgibbons 1997; Benichov et al. 2012), but as this research is based on measurements taken after a target word is encountered it does not necessarily indicate prediction processes (i.e., that a target word was preactivated and held in mind while concurrently comprehending ongoing speech). Although it is possible that prediction mechanisms were being employed in such research, the findings from these studies can as easily be explained by integration, that is, a contextually constrained word may simply have been easier to process once encountered because it fit better with the context (rather than that word being preactivated). We argue that no published research has directly investigated semantic prediction in PwHL, in terms of preactivation of a target before it has been heard (Fernandez et al. 2024b). Yet given the research outlined earlier, there are reasons to believe that PwHL may differ in their use of predictive mechanisms relative to PwNH (also see Fernandez et al. 2024b). In other words, while PwHL may particularly benefit from using top-down predictive mechanisms in the face of a degraded acoustic signal, they may not have the capacity to do so. Furthermore, if hearing loss leads to frequent mishearing, predictions may more often be inaccurate, decreasing the value of generating predictions in the first place (i.e., utility; Kuperberg & Jaeger 2016).

In this article, we present a VWP experiment investigating the timecourse of predictions of older adults with normal hearing versus postlingual hearing loss in sentences varying in contextual constraint. Across two sub-experiments, we investigate two different prediction stages: early agent-related associative predictions, and later narrowing of such predictions based on verb information. We hypothesize that hearing loss, particularly when accompanied by increased demand (and potentially, effort), will lead to delayed prediction, specifically for nonautomatic, resource-intensive prediction processes. Furthermore, we investigate whether the cost of predictions being violated varies between groups, as hearing loss could alternately decrease reliance on prediction (due to more frequent inaccuracy from mishearing), or increase reliance on prediction (due to poorer bottom-up processing).

## OVERALL DESIGN

This VWP study includes two sub-experiments, as well as fillers, interleaved within a single session. The sub-experiments differed in terms of the ease of predicting the intended target: whereas sub-experiment 1 used sentences in which both agent and verb constrained one possible target, sub-experiment 2 used sentences in which the agent constrained two competitors that were only narrowed to a single target by the verb. Filler sentences without constraint were included to enable analysis of any sentence processing differences that were not due to the use of prediction. This study was run with 3 groups of older adults: 1 with normal hearing (PwNH), 1 with hearing loss listening with low demand (due to speech being presented at a set level of 70 dB; PwHL_low-demand_), and 1 with hearing loss listening with high demand (due to speech level being individually adjusted in 5 dB steps to the lowest necessary for >70% intelligibility; PwHL_high-demand_). Note that we selected a 70% intelligibility threshold to increase demand while ensuring audibility remained high (and hence prediction possible). Full details in Procedure and Matching Between Groups. Differences in intelligibility and presentation level were confirmed by objective assessments, and differences in self-reported effort were also assessed (see Matching Between Groups). All data, code, and analyses are available on OSF (https://osf.io/ht9yn/).

## MATERIALS AND METHODS

### Participants

A total of 93 participants between 53 and 80 years old were included from the Hearing Sciences – Scottish Section participant database, mostly recruited via a local audiology clinic. We measured pure-tone thresholds according to BSA standards (British Society of Audiology 2018), and used their four-frequency pure-tone average (FFPTA) to categorize hearing group. For the PwNH group, we report the worse ear FFPTA, and for the PwHL groups, the better ear FFPTA. The PwNH group included 30 participants (FFPTA_mean_ = 14.66 dB HL, FFPTA_SD_ = 6.21, age_mean_ = 68.87 years, age_SD_ = 5.86 years). The PwHL groups included 63 participants (FFPTA_mean_ = 46.77 dB HL, FFPTA_SD_ = 9.35, age_mean_ = 72.32 years, age_SD_ = 5.18 years), who were randomly allocated to either the low demand group (n = 32) or the high demand group (n = 31). If participants used hearing aids they were asked to remove them during the study. Groups differed in the presentation level of the stimuli, Speech Intelligibility Index (ANSI S3.5 1997), intelligibility, and also self-reported effort (see Matching Between Groups). A total of 13 participants were excluded for technical reasons (n = 7), failure to complete the study (n = 4), or poor intelligibility (<50%, n = 2). See Table 1 for additional information. The sample size was based on an eye-tracking study investigating semantic prediction that found 20 to 30 participants with 16 items per condition was sufficient to obtain 80% power (using two different power analyses; Prystauka et al. 2024).

**TABLE 1.** Participant information

	PwNH	PwHL_low-demand_	PwHL_high-demand_
N (M/F)	30 (16/14)	32 (16/16)	31 (12/19)
Age (SD)	68.87 (5.86) PwNH-PwHL_low-demand_, *p* = <0.05* PwNH-PwHL_high-demand_, *p* = <0.05* PwHL_low-demand_-PwHL_high-demand_, *p* = 0.97	72.28 (5.18)	72.45 (5.27)
Better ear average dBHL (SD)	14.66 (6.22) PwNH-PwHL_low-demand_, *p* < 0.001* PwNH-PwHL_high-demand_, *p* < 0.001* PwHL_low-demand_-PwHL_high-demand_, *p* = 0.96	46.36 (7.83)	46.69 (10.56)
Presentation level dB (SD)	70 PwNH-PwHL_high-demand_, *p* < 0.001* PwHL_low-demand_-PwHL_high-demand_, *p* < 0.001*	70	64.68 (6.05)
Effort construct (SD)†	−0.72 (1.11) PwNH-PwHL_low-demand_, *p* = 0.13 PwNH-PwHL_high-demand_, *p* = <0.05* PwHL_low-demand_-PwHL_high-demand_, *p* = 0.21	−0.18 (1.40)	0.88 (2.64)
Speech Intelligibility Index	0.92 (0.04) PwNH-PwHL_low-demand_, *p* < 0.001* PwNH-PwHL_high-demand_, *p* < 0.001* PwHL_low-demand_-PwHL_high-demand_, *p* = 0.086	0.53 (0.17)	0.45 (0.16)
TRT score (SD)	58.47 (4.24) No differences	59.82 (3.13)	60.52 (5.04)
SicSpan WM score (SD)	19.60 (6.55) No differences	18.34 (6.37)	18.71 (5.81)
SicSpan intrusion score (SD)	3.20 (2.72) No differences	2.75 (2.14)	2.71 (1.94)
Intelligibility score	100(0) PwNH-PwHL_low-demand_, *p* = 0.086 PwNH-PwHL_high-demand_, *p* < 0.01* PwHL_low-demand_-PwHL_high-demand_, *p* < 0.05*	96.85 (11.48)	88.38 (20.50)

* Significant difference.

† The effort rating questionnaire consisted of six questions, and inspection of their correlation matrix revealed responses to be correlated with one another (*r*s > 0.40). Therefore, a principal component analysis was conducted to derive one underlying effort rating construct.

PwHL, people with hearing loss; PwNH, people with normal hearing.

Ethical approval was obtained from the University of University of Notthingham Faculty of Medicine and Health Science Research Ethics Committee (REC reference: FMHS 423-1221). Participants provided written informed consent to participate and were compensated £20.

### Apparatus

Eye movements were recorded with an Eyelink 1000 Plus, which was sampled at 1000 Hz, and the study was programmed using Experiment Builder. The head was stabilized using a chin rest and only the right eye was recorded. Participants sat approximately 80 cm from the screen. Stimuli were presented on a 17” ADI MicroScan A715 (Model Q17) monitor with 1024 × 768 resolution with 60 Hz refresh rate.

Audio materials were presented diotically via circumaural headphones (AKG reference 7202). While intensity was not root-mean-square normalized, variance was randomized across stimulus sets. Calibration was performed using a sound level meter, and daily headphone checks were conducted by the experimenter.

### Procedure

First, participants underwent air conduction audiometry, followed by an intelligibility check to determine the presentation level for the PwHL_high-demand_ subgroup and assess speech intelligibility for all groups. In this intelligibility check, participants were asked to repeat back a series of prerecorded sentences spoken by the talker of the later VWP stimuli (giving a best guess if necessary), and keyword accuracy was scored. For both the PwNH and PwHL_low-demand_ groups, a set of 5 sentences† was presented at 70 dB. For the PwHL_high-demand_ group, a first set of 5 sentences was presented at 60dB, and keyword accuracy was assessed, after which the presentation level was progressively increased in 5dB steps until over 70% keywords were correct (with different sentences being used at each level).

Next, participants completed the VWP experiment. This began with a standard nine-point calibration and validation of the right eye, followed by a practice section comprising three sentences. The visual world experiment proper consisted of 104 experimental trials presented in 2 blocks of 52. Sub-experiment 1 consisted of 48 items, sub-experiment 2 consisted of 16 items, and fillers made up the remaining 40 stimuli. Stimuli from both sub-experiments and fillers were evenly distributed across blocks, with two pseudo-randomized counterbalanced versions of the study being generated. Participants were told that they would hear sentences while viewing an array of objects and that their task was to listen for comprehension and subsequently identify the object that best matched the sentence just heard (using the mouse to click the relevant image). The trial began by displaying the image array for 2000 ms, after which the auditory stimulus was played while the image array remained displayed. A total of 2000 ms after the end of the auditory stimulus a green border appeared around the array, as well as a mouse pointer in the center of the screen allowing participants to click their selected image. A drift correct occurred between trials, a recalibration occurred between blocks, and eye movements were monitored throughout the experiment. Once the VWP was completed, participants responded to an effort questionnaire, specifically the National Aeronautics and Space Administration Task Load Index (NASA TLX) (Hart & Staveland 1988).

After the VWP, an unrelated phrase-end detection experiment was conducted (involving a combined button press and speech production task to investigate the relationship between temporal prediction and verbal turn-taking), which will be reported separately (Corps et al. submitted). Next, we included the Text Reception Threshold task (Zekveld et al. 2007) which measures the amount of visual information required to correctly recognize a written sentence, and finally a complex working memory span task interspersing size judgments with to-be-remembered items presented as text (Size-Comparison Span task: SiC Span) to assess working memory and semantic intrusion (Sörqvist et al. 2010).

### Matching Between Groups

Groups did not significantly differ on any of our cognitive measures (*p*s > 0.16; Table 1). However, they did slightly differ in age (*H*(2) = 8.16; *p* < 0.05), with PwNH being approximately 3.5 years younger than either PwHL group (PwNH = 68.87 PwHL_low-demand_ = 72.18 PwHL_high-demand_ = 72.45 *p*s < 0.05)‡.

As anticipated, the PwHL_high-demand_ group listened at a lower level and had a lower Speech Intelligibility Index (assessed with speech at the level the stimuli were presented in the experiment) than the PwNH group (*p* < 0.01). The PwHL_low-demand_ group listened at the same level and had a numerically lower Speech Intelligibility Index than the PwNH group though it did not reach significance (*p* = 0.086). We also analyzed self-reported effort ratings, and found that the PwHL_high-demand_ group reported significantly greater effort than the PwNH group (Wilcoxon rank sum test, *p* < 0.05), but no difference between the PwHL_low-demand_ and PwNH groups.

## SUB-EXPERIMENT 1

In sub-experiment 1, we tested the timecourse of predictions for the PwNH and PwHL groups and also investigated how they recover from a violated prediction (taken from Fernandez et al. 2024a, see OSF for complete list of items). To this end, we used the VWP with two sentence types: a constraining context culminating in a predictable target word (CP, e.g., *The tailor trims the suit*), and a constraining context culminating in an unpredictable but plausible target word (CU, e.g., *The tailor trims the tree*).

In terms of the timing of predictions, we analyzed when listeners looked to the predictable target in the CP items in relation to when the target was spoken. We hypothesized that PwNH would make use of contextual information to look to a target (*suit*) before it was explicitly spoken, but that hearing loss may delay this prediction because of its demand on cognitive resources. The behavior of low- and high-demand hearing loss groups was measured to allow investigation of whether any differences were driven by hearing loss itself or by consequent increases in demand (potentially leading to reduced availability of cognitive resources/increased listening effort).

In terms of recovery from violated predictions, we analyzed how quickly listeners switched to look at the (correct) unpredictable target in the CU items. Although listeners first prioritize the CP target due to its greater likelihood given the sentential constraint, the time it takes to switch to the CU target once heard provides an index of reliance on/difficulty overriding initial (incorrect) predictions. In terms of differences between groups, we considered two alternatives. On one hand, hearing loss could lead listeners to rely less on prediction, if mishearing leads to predictions being built on incorrect information and thus being inaccurate themselves. On the other hand, hearing loss could lead listeners to rely more on prediction due to poorer bottom-up processing if greater benefit (and reduced effort) could be attained through forward modeling than through bottom-up interpretation of a degraded signal.

### Materials and Methods (Sub-Experiment 1)

#### Stimuli

Twenty-four novel pairs of sentences were developed (Table 2). These were generated from a sentence frame with high constraint that ended either with a predictable target (CP) or an unpredictable target (CU). The sentences followed the structure “The [agent] [verb] the [object].” Thus, the CU items had the same contextual information as the CP, but the final target word was not the predictable object, rather the target was unpredictable but a plausible and appropriate object of the verb. Stimuli were presented in two blocks, each containing 12 of each item type (CP/CU). See Table 2 for example items, the Zipf frequency§ of the agent, verb, and critical word, and the mean syllable count per word.

**TABLE 2.**
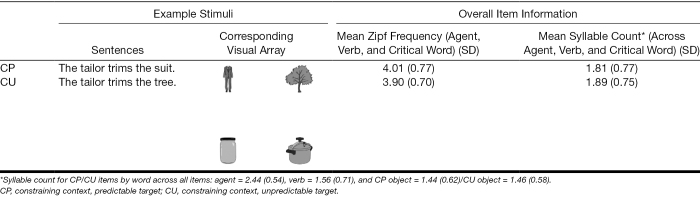
Sub-experiment 1 example stimuli and item information

Each sentence pair had a corresponding visual array consisting of four images: the CP target (*suit*), the CU target (*tree*), and two distractor items that were not compatible with the CP/CU verb (i.e., in the following example, they are not trimmable; *jar* and *pot*). Therefore, an item consisted of one sentence and the four corresponding images. All images were grayscale 300 × 300 pixel jpeg line drawings taken from the MultiPic which have been normed for British English (Duñabeitia et al. 2018), and no images in the same array shared initial phonemes. Placements of the images were rotated such that each item occurred in each location 25% of the time. According to the SUBTLEX-UK database, the frequencies of the image names ranged from medium to high (M = 4.10, SD = 0.73). See Appendix A, Supplemental Digital Content (http://links.lww.com/EANDH/B663) for item pretesting information.

Sentences were recorded by a male native speaker of Scottish English (i.e., the accent relevant for our participant population) using Audacity recording software (Audacity Team 2021) with a Blue Yeti USB microphone at a 48,000 Hz sampling rate. For ease of post-hoc manipulation (i.e., to ensure all trials were a consistent duration), a click track at 90 bpm was used to aid the speaker in producing words with different syllable counts within the same time frame. Using Praat (Boersma & Weenink 2021), the mean duration of the corresponding word across all sentences was found (i.e., separately for The, [Agent], [Verb], The, and [Object]), and this mean used to normalize the word across all sentences, thus each corresponding word was the same duration across all items (normalized word durations [ms]: The = 93.58, [Agent] = 612.72, [Verb] = 602.05, The = 130.27, and [Object] = 464.45). The speech rate across items ranged from 2.56 to 5.65 syllables per second with a mean of 3.47 (SD = 0.77) syllables per second.

#### Analysis

We analyzed the accuracy with which participants selected the target after the sentence finished (i.e., clicking on the image corresponding to the final word) as well as timing of looks to targets while listening. Note that we ran each analysis with the full participant set (described later), and then again with only those participants scoring 100% on the intelligibility pretest to ensure lack of audibility did not drive our reported effects (thus removing 0 PwNH, 2 PwHL_low-demand,_ and 10 PwHL_high-demand_). Results remained largely unchanged, with any differences reported in footnotes. Please see OSF for data, additional visualizations, and complete analysis (across all participants as well as the subset of 100% intelligibility participants).

##### Accuracy

Whether participants clicked on the correct item at the end of each sentence was analyzed to ensure that they comprehended the sentence appropriately. Accuracy was coded as 1 (correct) or 0 (incorrect) and submitted to a logit mixed model. Fixed effects for the accuracy measure included sentence type (CU, CP) which was sum contrast coded (0.5/−0.5) and group (PwNH, PwHL_low-demand_, PwHL_high-demand_) which was treatment contrast coded (with PwNH serving as the baseline). Random effects of participant and item were included and maximally specified (Barr et al. 2013)¶. The maximal model did not converge therefore the random effects structure was simplified by removing the interaction in the random effect of item (to main effects) and convergence was reached∥. An omnibus test for main effects and interaction was run using a log-likelihood ratio test, and only contrasts from comparisons reaching significance are interpreted.

##### Divergence Point Analysis

The timing of looks to the objects across conditions and between groups was investigated using a divergence point analysis (DPA; Stone et al. 2021). DPA is a nonparametric bootstrapping analysis that estimates the time at which looks to one object diverge from looks to another object. A divergence point is determined by comparing the proportion of looks between the two objects across a time window (see below for specific comparisons) and finds the time in which the objects significantly differ across 10 consecutive 20 ms time bins using *t* tests (i.e., over 200 ms). Using nonparametric bootstrapping, 2000 new data sets are generated by sampling across participant, object, time, and group and a mean divergence point and confidence interval (CI) is estimated. The DPA is an ideal measure because it deals with the inherent nonindependency of fixations while decreasing the likelihood of type 1 error and estimating CIs, thus allowing us to estimate effect latencies between two groups as well as the uncertainty around the effect (which is not possible using other approaches such as cluster-based permutations, general additive mixed models, or growth curve analyses). Therefore, differences between groups will be based on CIs as opposed to more traditional statistical tests based on *p* values (see Stone et al. (2021) for additional information about DPA and Ito & Knoeferle (2023) for a comparison of statistical approaches to VWP data). See OSF for complete data and code.

We ran DPA analyses on only those items that were correctly answered (see below for more information about accuracy). Regions of interest were defined around the four images in the array (300 × 300 pixels) with an additional 50 pixels on each side. The first DPA analysis, which we refer to as the prediction analysis, was to test the timecourse of prediction across the three groups by investigating when they looked to the CP target (*suit*) while hearing *The tailor trims the suit*. If looks to the CP target diverge from non-target images before the onset of the CP target we take this as evidence of prediction. To be as conservative as possible, we chose to compare looks to *suit* to the only other image that is an appropriate object for the verb, that is, the CU target *tree*. The second DPA analysis, which we refer to as the cost analysis, was to test the effect of violated predictions across our three groups by investigating how quickly listeners integrated an unexpected CU target (*tree*) when hearing *The tailor trims the tree*…. Comparing the CU target (which had originally been disregarded), and a distractor object should allow assessment of how quickly participants access and integrate *tree* once it is heard**.

### Results (Sub-Experiment 1)

#### Accuracy

For the CP items, the overall mean accuracy was 99% (all groups scored above 97%), and for the CU items, the overall accuracy was 92% (all groups scored above 88%). Note that the correct answer for the CP items is different than the correct answer for the CU items. The omnibus test revealed a main effect of type (*X*^2^(1) = 26.86; *p* < 0.001) with CU items having a lower accuracy than CP items, but no significant difference by group (Table 3).

**TABLE 3.** Sub-experiment 1 accuracy model output and means

Fixed Effects					
Model Parameter		*b*	SE	*z*	*p*
Intercept		6.74	0.69	9.71	<0.001
PwNH–PwHL-low-demand		−1.12	0.82	−1.36	0.18
PwNH–PwHL-high-demand		−1.71	0.79	−2.16	<0.05*
Type		4.11	1.20	3.44	<0.001†
PwNH–PwHL-low-demand† type		−1.59	1.31	−1.21	0.23
PwNH–PwHL-high-demand† type		−1.55	1.23	−1.26	0.21

Random Effects					
		Variance	SD	Corr	
Participant (intercept)		2.69	1.64		
Type		1.17	1.08	−0.21	
Item (intercept)		0.95	0.98		
PwNH–PwHL-low-demand		1.75	1.32	−0.73	
PwNH–PwHL-high-demand		1.34	1.16	−0.62	
type		4.93	2.22	0.99	

Mean (SD) Comprehension Accuracy: Sub-Experiment 1					
Type	PwNH	PwHL-Low-Demand		PwHL-High-Demand	
CP	99.72 (5.27)	99.35 (8.05)		98.52 (12.08)	
CU	95.42 (20.93)	92.97 (25.58)		89.65 (30.48)	

* Given that the omnibus test of group do not reach significance, we do not interpret this finding.

† Significant difference.

CP, constraining context, predictable target; CU, constraining context, unpredictable target; PwHL, people with hearing loss; PwNH, people with normal hearing.

**Fig. 1.**
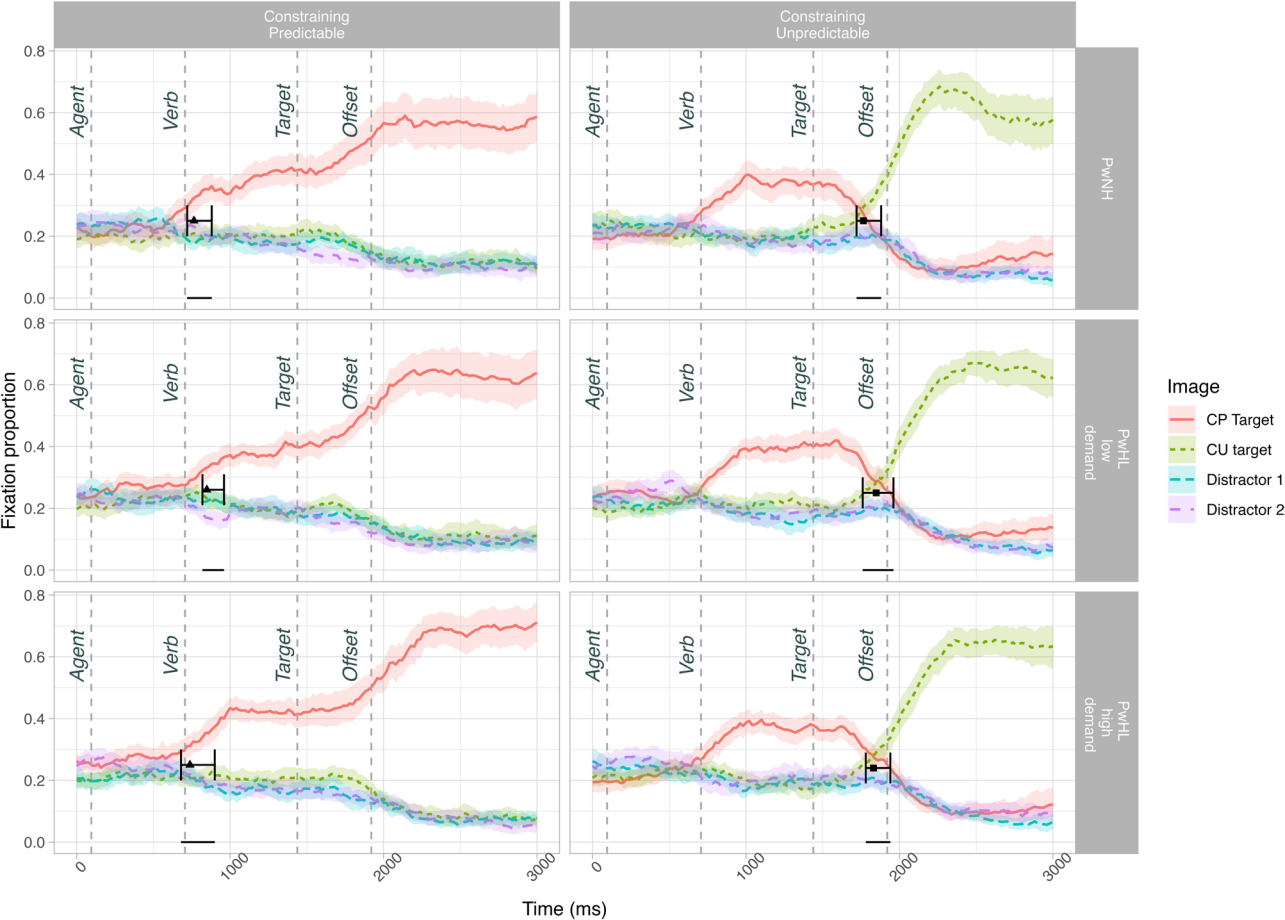
Fixation proportion of correctly answered items to all objects across sentence types in sub-experiment 1. As the items were manipulated to ensure that word timings were the same, the dashed vertical lines depict the onset of each word. The prediction analysis divergence point (indicated with a triangle) and 95% confidence intervals are superimposed on the CP items and compare the fixation proportion of looks to the CP target and CU target. The prediction cost analysis divergence point (indicated with a square) and 95% confidence intervals is superimposed on the CU items and compare the fixation proportion of looks to the CU target and distractor 1. The horizontal dash on the 0 line indicates the 95% confidence interval window. CP indicates constraining context, predictable target; CU, constraining context, unpredictable target.

Incorrectly answered items were removed from the DPA analysis (4.08%). See Figure 1 for fixation proportions to each object across time for correctly answered items.

#### Divergence Point Analysis

The prediction analysis compared looks to the CP target (*suit*) relative to looks to the only other appropriate object to the verb (i.e., the CU target *tree*) in the 24 CP items only (*The tailor trims the suit*; Fig. 1). The analysis revealed a divergence point for PwNH at 764.45 (CI: 720 to 880), for PwHL_low-demand_ at 848.42 (CI: 820 to 960), and for PwHL_high-demand_ at 738.17 (CI: 680 to 900). The divergence points did not differ between groups. The mean difference between the PwNH and PwHL_low-demand_ group is 83.97 ms (CI: −20 to 180)††, and given that the CI contains 0, we cannot conclude these groups differ. The mean difference between the PwNH and PwHL_high-demand_ group is −26.28 (CI: −140, 120), and given that the CI contains 0, we cannot conclude these groups differ.

The cost analysis compared looks to the CU target (*tree*) relative to looks to a distractor (*jar*) in 24 CU items only (*The tailor trims the tree*) (Fig. 1; see OSF for further visualizations). The analysis revealed a divergence point for PwNH at 1764.13 (CI: 1720 to 1880), for PwHL_low-demand_ at 1848.05 (CI: 1760 to 1960), and for PwHL_high-demand_ at 1829.96 (CI: 1780 to 1940). The divergence points did not differ between groups. The mean difference between the PwNH and PwHL_low-demand_ group is 83.92 ms (CI: −60 to 200), and given that the CI contains 0, we cannot conclude these groups differ. The mean difference between the PwNH and PwHL_high-demand_ group is 65.83 (CI: −60 to 180), and given that the CI contains 0, we cannot conclude these groups differ.

### Discussion (Sub-Experiment 1)

In sub-experiment 1, we used the VWP to test whether age-matched PwNH and two groups of PwHL were able to use contextually constraining information to make associative predictions during listening, and if they were able to inhibit predictions and rapidly integrate unexpected targets when predictions were not met. We tested two sentence types: a constraining context with a predictable target word (CP, e.g., *The tailor trims the suit*), and a constraining context with an unpredictable but plausible target word (CU, e.g., *The tailor trims the tree*). We hypothesized that PwNH would show predictive looks to the CP target, but that hearing loss, or the increased listening difficulty caused by hearing loss, may delay prediction. In relation to violated predictions, we speculated that PwHL may either show less reliance on prediction due to greater inaccuracy or greater reliance on prediction due to poorer bottom-up processing.

We found that all groups made predictions, that is, looked to the predictable target before hearing it, and that there were no differences in the timing of these predictions between groups. The onset of the target word occurred at approximately 1440 ms, and all three groups showed a divergence between the CP target (*suit*) and CU target (*tree*) in the CP items (*The tailor trims the suit*), at 760 to 850 ms. As the verb onset occurred at approximately 700 ms, this suggests that all groups were able to predict soon after the onset of the verb. In addition, no group differences were found in the cost analysis, which investigated the timing of looks to the unexpected (but ultimately correct) target (*tree*) relative to a distractor object (*jar*) in the CU items (*The tailor trims the tree*). As the target word was spoken between 1440 and 1780 ms, and all groups showed a divergence to the target relative to the distractor between 1760 and 1850 ms, this suggests that participants were able to inhibit their original prediction to integrate the unexpected target (*tree*) within a few hundred milliseconds of hearing it.

Together these data suggest that older adults with and without hearing loss are able to use context to make predictions, and, if a prediction is made but not met, integrate the correct word soon after disambiguating phonological information is provided. In spite of the potential costs associated with prediction (Huettig & Janse 2016; Ito et al. 2017; Nitsan et al. 2019, 2022; Harel-Arbeli et al. 2023), hearing loss (George et al. 2007; Carroll et al. 2016), and degraded listening (Pichora-Fuller et al. 2016; Peelle 2018), sub-experiment 1 provides clear evidence that predictive mechanisms remain functional in PwHL. Furthermore, the seeming lack of difficulty integrating unexpected targets across these groups suggests that listeners may activate broad lexical information when making predictions which makes integration of an unexpected target relatively cost-free (Luke & Christianson 2016; Frisson et al. 2017; Chow & Chen 2020; Fernandez et al. 2024a).

Importantly, while we see strong prediction effects in all groups in sub-experiment 1, we note that the stimuli we used were particularly simple, (i.e., requiring minimal resources to process and predict). To further explore predictive processing with hearing loss and listening demand, in sub-experiment 2, we tested the timecourse by which predictions are built up, investigating how semantic competition is disambiguated with progressive contextual information.

## SUB-EXPERIMENT 2

In sub-experiment 2, we investigated how predictions are modified over time, using the same participants as in sub-experiment 1, particularly testing the different stages of prediction. The first stage of prediction is argued to be early, automatic, quick, less costly, and rooted in lexical priming, while the second stage is argued to be later, non-automatic, slower, more resource-demanding, and require real-word knowledge (Pickering & Gambi 2018; Corps et al. 2022). Although the first stage is quick, it is also error prone (as associated concepts may become activated that do not fit into the sentence context; Kukona et al. 2011). The second stage is later and more resource-demanding because it requires pruning the prediction to the context (e.g., inhibiting inappropriate activations and tailoring the prediction to the situation; Corps et al. 2022). In this sub-experiment, we used a different set of items to assess how listeners combine agent and verb information as context unfolds. Specifically, the agent in these stimuli activated two competing images which were narrowed to a single target by the verb, meaning that listeners could use the verb restriction information to facilitate the relevant target (and inhibit the competitor). If the prediction demonstrated in sub-experiment 1 was based on spreading activation (i.e., an automatic and unconscious process), we anticipated that hearing status and listening demand would specifically impact the more resource-demanding nonautomatic second stage of tailoring predictions.

Given that we found clear evidence of prediction in all groups in sub-experiment 1, we first hypothesized that we would also see early agent-based prediction in this experiment through a preference for the two potential targets relative to the distractors, with no differences between groups. Second, we hypothesized that we would however see delays for PwHL relative to PwNH in relation to how early listeners prioritized the target over the agent-related competitor once the verb occurred, due to the more resource-demanding process of prediction adjustment. Depending on whether effects were due to hearing loss itself, or the indirect (potentially cognitive) effects of increased listening demands, we expected to see delays in prediction either between the PwNH group and both PwHL groups or confined to the PwHL_high-demand_ group alone.

### Materials and Methods (Sub-Experiment 2)

#### Stimuli

The items for sub-experiment 2 consisted of 16 contextually constraining sentences (Table 4) with a predictable target word (CP). These followed the same structure as sub-experiment 1 (i.e., The [agent] [verb] the [critical word]), and are almost identical to those used by Holt et al. (2021)‡‡, with slight adjustments based on the norming procedure for our specific population (Appendix A, Supplemental Digital Content, http://links.lww.com/EANDH/B663). Stimuli were presented in two blocks, each containing eight items. See Table 4 for example items, the Zipf frequency of the agent, verb, and critical word, and the mean syllable count per word.

**TABLE 4.**
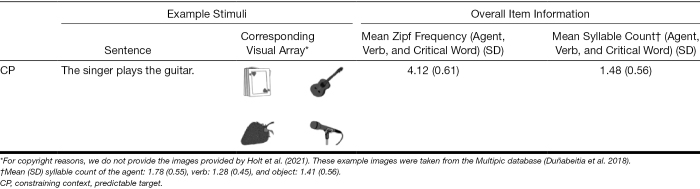
Sub-experiment 2 example stimuli and item information

Each sentence had a corresponding visual array consisting of four images provided by Holt et al. (2021): the CP target (*guitar*), an agent-related image (unrelated to the verb; *microphone*), a verb-related image (unrelated to the agent; *cards*), and a distractor (*strawberry*). All images were grayscale 300 × 300 pixel jpeg line drawings which were pre-tested with older adult British-English speakers, and no images in the same array shared initial phonemes. Placements of the images were rotated such that each item occurred in each location 25% of the time. According to the SUBTLEX-UK database, the frequencies of the image words ranged from medium to high (M = 4.14, SD = 0.61). For pretesting information, see Appendix A, Supplemental Digital Content (http://links.lww.com/EANDH/B663).

The sentences were recorded and mean normalized as outlined in sub-experiment 1.

#### Analysis

As in sub-experiment 1, accuracy and timing were analyzed and also replicated with only those participants scoring 100% intelligibility on the intelligibility pretest (thus removing 0 PwNH, 2 PwHL_low-demand,_ and 10 PwHL_high-demand_, any differences reported in footnotes).

##### Accuracy

Accuracy was conducted as outlined in sub-experiment 1. The model was fit with one fixed effect of group (PwNH, PwHL_low-demand_, PwHL_high-demand_) which was treatment contrast coded (with PwNH serving as the baseline). Random effects of participant and item were included and maximally specified (Barr et al. 2013)§§.

##### Divergence Point Analysis

We ran two prediction DPA analyses on those items that were correctly answered to test prediction across the three groups. The first DPA was similar to the prediction DPA of sub-experiment 1, and compared looks to the target (*guitar*) relative to the verb-related distractor (*cards*) during the predictable context item (*The singer plays the guitar*). The second prediction DPA differed importantly from the former by addressing the time at which the predictable competitors were disambiguated, specifically by comparing looks to the predictable target (*guitar*) relative to the agent-related competitor (*microphone*).

### Results (Sub-Experiment 2)

#### Accuracy

Overall mean accuracy was 97.44% (all groups scored above 95%). The omnibus test revealed no main effect (*p* > 0.30; see OSF; Table 5).

**TABLE 5.** Sub-experiment 2 accuracy model output and means

Fixed Effects				
Model Parameter	*b*	SE	*z*	*p*
Intercept	12.30	2.04	6.02	<0.001
PwNH–PwHL-low-demand	−0.72	2.49	−0.29	0.77
PwNH–PwHL-high-demand	−3.46	2.64	−1.31	0.19

Random Effects				
	Variance	SD	Corr	
Participant (intercept)	2.69	1.64		
Item (intercept)	1.17	1.08	−0.21	
PwNH–PwHL-low-demand	0.95	0.98		
PwNH–PwHL-high-demand	1.75	1.32	−0.73	

Mean (SD) Comprehension Accuracy: Sub-Experiment 2				
Type	PwNH	PwHL-Low-Demand	PwHL-High-Demand	
CP	98.33 (12.82)	97.27 (16.32)	96.77 (17.69)	

CP, constraining context, predictable target; PwHL, people with hearing loss; PwNH, people with normal hearing.

**Fig. 2.**
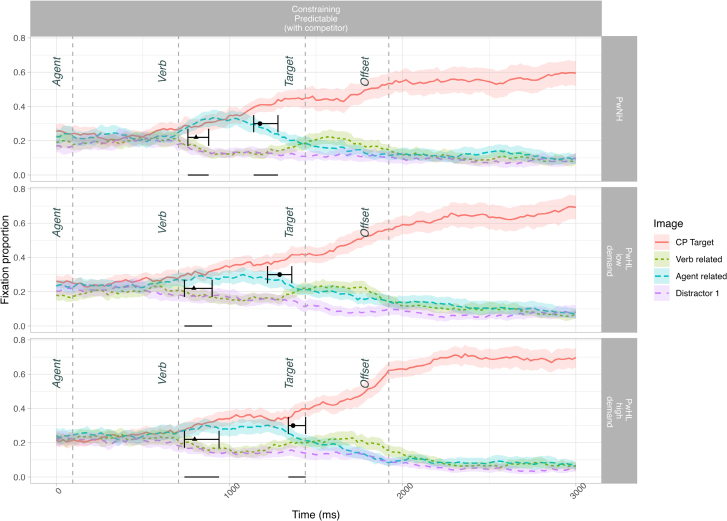
Fixation proportion of correctly answered items to all objects across sentence types in sub-experiment 2. (It is interesting that although non–agent-related items [i.e., the verb-related cards, and distractor strawberry] should have been discarded as soon as the agent was heard, visual inspection suggests increased looks to the verb-related image after the verb was spoken [i.e., a temporary increase in looks around the start of the target in this figure]. This was particularly evident for PwNH and PwHL_low-demand_. While inappropriate in the larger context, such looks to the verb-related image have been previously reported in college-aged adults, second language speakers, and children [Kukona et al. 2011; Borovsky et al. 2012; Peters et al. 2018], and been proposed to stem from thematic priming [Ferretti et al. 2001]. While it is unclear what is driving this in our groups, it is relatively clear that there is some verb-driven activation occurring that may differ across groups, and may be a compelling line for future research.) The early-stage divergence point and 95% confidence intervals (indicated with a triangle) are superimposed on the CP items and compare the fixation proportion of looks to the CP target and verb-related image. The late-stage divergence point and 95% confidence intervals (indicated with a circle) are superimposed on the CP items and compare the fixation proportion of looks to the CU target and agent-related image. The horizontal dash on the 0 line indicates the 95% confidence interval window. CP indicates constraining context, predictable target; CU, constraining context, unpredictable target; PwHL, people with hearing loss; PwNH, people with normal hearing.

Incorrectly answered items were removed from the DPA analysis (2.56%). See Figure 2 for fixation proportions to each object across time for correctly answered items.

#### Divergence Point Analysis

The first prediction analysis compared looks to the CP target (*guitar*) relative to looks to the verb-related distractor (*cards*) in the CP items (*The singer played the guitar*). The analysis revealed a divergence point for PwNH at 807.36 (CI: 760 to 880), for PwHL_low-demand_ at 794.60 (CI: 740 to 900), and for PwHL_high-demand_ at 799.15 (CI: 740 to 940). The divergence points did not differ between groups. The mean difference between the PwNH and PwHL_low-demand_ group is −12.76 ms (CI: −120 to 120), and given that the CI contains 0, we cannot conclude these groups differ. The mean difference between the PwNH and PwHL_high-demand_ group is −8.21 ms (CI: −100 to 120), and given that the CI contains 0, we conclude that these two groups do not differ (Fig. 2; see OSF for further visualizations). This replicated the findings from sub-experiment 1.

The second prediction analysis compared looks to the CP target (*guitar*) relative to looks to the agent-related competitor (*microphone*). The analysis revealed a divergence point for PwNH at 1176.13 (CI: 1140 to 1280), for PwHL_low-demand_ at 1289.83 (CI: 1220 to 1360), and for PwHL_high-demand_ at 1367.56 (CI: 1340 to 1440). The divergence point for PwNH was earlier than either PwHL group. The mean difference between the PwNH and PwHL_low-demand_ group is 113.70 ms (CI: 0 to 200)¶¶, and given that the lower bound of CI is 0, we conclude that divergence point for PwNH is marginally earlier relative to the PwHL_low-demand_. The mean difference between the PwNH and PwHL_high-demand_ group is 191.43 (CI: 100 to 280), and given that the CI does not contain 0, we conclude that the divergence point for PwNH is significantly earlier than PwHL_high-demand_ (Fig. 2).

### Discussion (Sub-Experiment 2)

In sub-experiment 1, we found that both PwNH and PwHL were able to use contextual information to make early associative predictions within a similar time frame. In sub-experiment 2, we further investigated the timecourse of prediction by distinguishing between the initial activation of predictable items, and a second stage of tailoring the array of activated predictable items to the context as it unfolded. We tested predictable context items (e.g., *The singer plays the guitar*) with four image types: target (*guitar*), an agent-related image (*microphone*), a verb-related image (*cards*), and an unrelated distractor image (*strawberry*). We first compared looks to the target versus the verb-related distractor (initial activation of agent-related predictions as in sub-experiment 1) and second compared looks to the target vs. the agent-related competitor (narrowing of predictions with context). We proposed that whereas automatic spreading activation may underlie early predictions, incremental tailoring of predictions may be a more strategic process requiring greater cognitive resources.

Replicating findings from sub-experiment 1, we found that both PwNH and PwHL made agent-related predictions, in that they were looking to the target (*guitar*) relative to the verb-related object (*cards*) before it was spoken. This early prediction did not differ between groups, suggesting an automatic and less costly process. Strikingly however, we found graded differences between the groups in terms of when they were able to narrow predictions to discard the competitor: the divergence for the PwHL_low-demand_ group was marginally later than the PwNH group, and the PwHL_high-demand_ was significantly later than the PwNH. Thus, hearing loss delays tailoring of predictions relative to PwNH, with greater listening demands exacerbating this difference, and these findings suggest that such second-stage predictions are nonautomatic and resource-intensive.

## EXPERIMENTAL FILLERS

Finally, we included neutral context sentences to provide information about when different groups access lexical information in the absence of prediction. These sentences do not provide contextual constraint to enable prediction and thus looks to the target can only occur after enough phonological information has been received from the target word.

### Materials and Methods (Experimental Fillers)

#### Stimuli

The fillers consisted of 40 neutral context sentences that followed the same structure as the experimental sentences described in sub-experiments 1 and 2 (i.e., The [agent] [verb] the [critical word]), and included the same target object. Stimuli were presented in two blocks, each containing 20 items. See Table 6 for an example item, the Zipf frequency of the agent, verb, and critical word, and the mean syllable count per word.

**TABLE 6.**
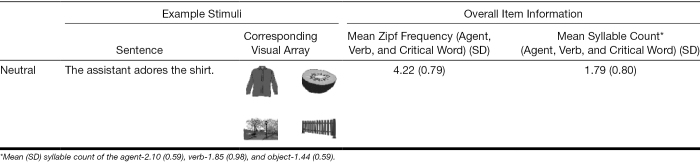
Filler sentence example stimuli and item information

Each sentence had a corresponding visual array consisting of four images: the target (*shirt*) and three distractors (*park, kiwi, fence*). All images were grayscale 300 × 300 pixel jpeg images, taken from either the MultiPic or from Holt et al. (2021), and the placements of the images were rotated such that each item occurred in each location 25% of the time. According to the SUBTLEX-UK database, the frequencies of the image words ranged from medium to high (M = 4.22, SD = 0.78). For pretesting information see, Appendix A, Supplemental Digital Content (http://links.lww.com/EANDH/B663).

The sentences were recorded and mean normalized as outlined in sub-experiment 1.

#### Analysis

As in sub-experiments 1 and 2, analyses were conducted as described in the following paragraph and replicated with only those participants scoring 100% intelligibility.

##### Accuracy

Accuracy was conducted as outlined in sub-experiment 2. Random effects of participant and item were included and maximally specified (Barr et al. 2013)∥∥.

##### Divergence Point Analysis

DPA was used to analyze the timing of looks to the objects across groups as outlined in sub-experiment 1. We compare looks to the target (*shirt*) relative to a distractor (*kiwi*) in the neutral context items (*The assistant adores the shirt*).

### Results (Experimental Fillers)

#### Accuracy

Overall mean accuracy was 97.44% (all groups scored about 94%). The omnibus test revealed a main effect of group (*X*^2^(1) = 25.96, *p* < 0.001) with PwHL_low-demand_ surprisingly scoring higher than PwNH (*z* = 2.19, *p* = 0.03) and PwHL_high-demand_ scoring lower than PwNH (*z* = −2.53, *p* = 0.01)***; see OSF (mean accuracy: PwNH: 98.33%, PwHL_low-demand_ = 98.60%, PwHL_high-demand_ = 95.50%). Note however that accuracy differences were remarkably small (i.e., all accuracies were between 95 and 99%; Table 7).

**TABLE 7.** Fillers accuracy model output and means

Fixed Effects				
Model Parameter	*b*	SE	*z*	*p*
Intercept	6.06	0.57	10.56	<0.001
PwNH–PwHL-low-demand	2.07	0.95	2.19	<0.05*
PwNH–PwHL-high-demand	−1.72	0.68	−2.53	<0.05*

Random Effects				
	Variance	SD	Corr	
Participant (intercept)	2.44	1.56		
Item (intercept)	0.73	0.85		
PwNH–PwHL-low-demand	6.77	2.60	0.17	
PwNH–PwHL-high-demand	1.63	1.28	−0.63	

Mean (SD) Comprehension Accuracy: Fillers				
Type	PwNH	PwHL-Low-Demand	PwHL-High-Demand	
CP	98.33 (12.81)	98.59 (11.78)	95.4 (20.95)	

* Significant difference.

CP, constraining context, predictable target; PwHL, people with hearing loss; PwNH, people with normal hearing.

**Fig. 3.**
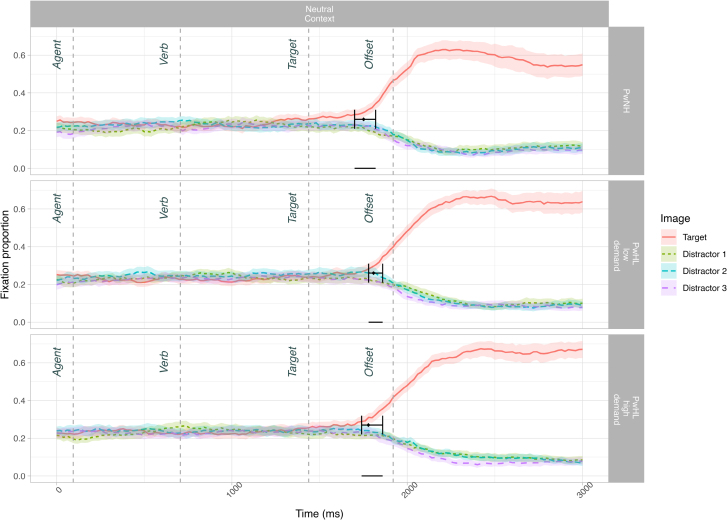
Fixation proportion of correctly answered items to all objects across filler sentences. The divergence point (indicated with a diamond) analysis and 95% confidence intervals are superimposed on the neutral items and compare the fixation proportion of looks to the target and distractor. The horizontal dash on the 0 line indicates the 95% confidence interval window.

Incorrectly answered items were removed from the DPA analysis (2.55%). See Figure 3 for fixation proportions to each object across time for correctly answered items.

#### Divergence Point Analysis

The analysis revealed a divergence point for PwNH at 1751.32 (CI: 1700 to 1820), for PwHL_low-demand_ at 1807.87 (CI: 1780 to 1860), and for PwHL_high-demand_ at 1778.64 (CI: 1740 to 1860) (Fig. 3). Surprisingly, the divergence point for PwNH was marginally earlier than the PwHL_low-demand_ group only. The mean difference between the PwNH and PwHL_low-demand_ group is 56.55 ms (CI: 0 to 140), and given that the lower bound of CI is 0, we conclude that divergence point for PwNH is marginally earlier relative to the PwHL_low-demand_. The mean difference between the PwNH and PwHL_high-demand_ group is 27.32 (CI: −60 to 120), and given that the CI contains 0, we cannot conclude that the divergence point for PwNH differs from the PwHL_high-demand_ group.

### Discussion (Experimental Fillers)

In our analysis of experimental fillers, we aimed to assess differences between groups in simple lexical access of heard words, based on listening to stimuli in which the sentence frame did not constrain any particular target. Although we did not see sizable differences in terms of when groups diverged, there was an unexpected and marginally significant difference between PwNH and PwHL_low-demand_. It is not clear why PwNH would diverge earlier than PwHL_low-demand_ but no differently from PwHL_high-demand_. However, note that the difference was small, being of the order of 50 ms, which is substantially smaller than the prediction differences in sub-experiment 2 (which were as great as 190 ms). Since the divergence time differences in sub-experiment 2 were greatest between the PwNH and PwHL_high-demand_ group, the lack of difference between PwNH and PwHL_high-demand_ in the neutral stimuli provides additional support that the effects reported earlier are prediction-specific, rather than a simple byproduct of delayed lexical access.

## GENERAL DISCUSSION

Age-related hearing loss negatively impacts speech perception and can lead to a greater reliance on the cognitive system to extract meaning (Peelle 2018), decreasing available resources for language tasks relative to PwNH (George et al. 2007; Carroll et al. 2016). Although semantic prediction can benefit listeners by increasing language processing accuracy, speed, and ease (as described by Holt et al. 2021), we hypothesized that hearing loss may in fact reduce the use of prediction to further compound speech listening difficulty (Fernandez et al. 2024b). Across two sub-experiments, we tested prediction using the VWP, addressing effects of hearing loss and related increases in listening demand by manipulating stimulus presentation level. In sub-experiment 1, we tested whether two groups of PwHL and a group of approximately age-matched PwNH were able to use contextually constraining information to make early associative predictions during listening, and resolve violated predictions when hearing an unexpected target. In sub-experiment 2, we used more complex items to investigate differences in how predictions are built up over time, by including competing images that could only be discarded using verb restriction information through later modification, potentially tapping into a more resource-demanding second stage of tailoring of predictions.

In sub-experiment 1, we found that PwNH and both groups of PwHL used contextual information to make early predictions about an upcoming object for simple sentences with a clear target. When hearing a constraining context, such as *The tailor trims the*, all three groups looked to the target object *suit* long before it was heard, and soon after the onset of the verb, with no differences in timing between groups. While previous research has shown the benefit of contextual information, this study provides the first direct demonstration of prediction in older adults with hearing loss (Fernandez et al. 2024b), and the lack of group difference indicates that not only did PwHL predict but that they predicted at a similar speed to PwNH. In addition, and unexpectedly, we found no evidence of hearing loss affecting reliance on contextual information when predictions were violated (Sommers & Danielson 1999; Rogers et al. 2012; Failes et al. 2020; Van Os et al. 2021; Failes & Sommers 2022), with all groups being able to quickly integrate incorrectly predicted targets. Previous research has consistently reported greater false hearing (or prioritization of prediction) in older than younger adults, without addressing the basis of this difference, and the lack of dependence of such an effect on hearing ability or listening demand in the present study was surprising. However, in prior work, the stimuli often comprised simple sentences that provided early constraint for a single likely target, then a phonological (possibly implausible) competitor being presented masked in noise. Such a paradigm may have increased the likelihood of mishearing (by both generating a strong prediction and poor evidence to its contrary). Our study, on the other hand, offered plausible targets, audible and unaffected by noise, and with no phonological overlap, reducing the chance of such an error. Overall, the prediction tapped in sub-experiment 1 may therefore stem from a relatively automatic and resource-free early stage of prediction that has little cost and is characterized by spreading activation (Pickering & Gambi 2018; Corps et al. 2022).

To address prediction in a more nuanced manner, sub-experiment 2 investigated how contextual constraints were progressively combined to tailor predictions, using sentences in which competitors could be differentiated at the verb. We again found that PwNH and both groups of PwHL were able to use contextual information to make early agent-related predictions, with no group differences in looks to the target over the verb-related distractor. It is important to note that, however, we did see group differences in the time at which the verb-related information was used to narrow these predictions (and discard the semantic competitor). Looks to the target over the semantic competitor diverged earlier for PwNH relative to PwHL, with PwHL_low-demand_ diverging marginally later (at 114 ms, becoming significant in the highly intelligible subset analysis) and PwHL_high-demand_ diverging significantly later (at 191 ms). This suggests that in the face of sentences with progressively narrowing contextual constraints, later-stage predictions are delayed by hearing loss, and further impacted by listening demand. Given we did not see comparable differences in the timing of processing neutral stimuli, these effects cannot be explained by simple differences in lexical access time.

Together, our findings indicate that older adults with and without hearing loss are able to use contextual information to make simple agent-based predictions. These early-stage predictions do not appear to be impacted by group differences and may be based on an automatic and cost-free process of spreading activation. However, when predictions need to be refined over time, we suggest that they may rely on a different, nonautomatic, and costly process (Pickering & Gambi 2018; Corps et al. 2022). It is this latter stage of prediction that we found to be impacted by hearing loss when listening at high demand. Note that while the PwNH group was slightly younger than the PwHL groups, all reported effects replicated when only PwNH over age 65 were included, indicating that these effects were not driven by age.

There are several reasons that PwHL may show delayed late-stage prediction relative to PwNH: high cost, reduced reliability, or use of alternative strategies. If the cost of prediction is too high for PwHL, it may be downweighted in favor of bottom-up processing. Alternatively, based on the idea of utility (Kuperberg & Jaeger 2016), if predictions are unreliable for PwHL (i.e., too frequently incorrect), the mechanism may be used less. Finally, PwHL may direct the cognitive resources that they do have available to alternative strategies such as reinterpreting previously heard contextual information (Gwilliams et al. 2018) or processing facial expressions or gestures for meaning. Disentangling these possibilities could provide substantial insight into the cognitive impacts of hearing loss, and could also contribute to forming a more comprehensive theoretical understanding of prediction more broadly.

In an attempt to address the potential basis of such prediction delay with hearing loss, we conducted an exploratory analysis of how differences in effort (i.e., cost), in the absence of differences in intelligibility, affected prediction timing. This analysis included only participants who scored 100% on the intelligibility test (N = 76), split into groups based on hearing status and subjective effort (<15/100 being low effort, and >15/100 being high effort). This led to 3 groups that were matched in intelligibility but differed in effort: 1 PwNH group (with a subjective effort rating <15) and 2 PwHL groups split into low-effort (with a subjective effort rating <15) and high-effort (with a subjective effort rating >15). The low- and high-effort PwHL groups had a lower presentation level to the PwNH but did not differ from each other (and differences were <4 dB). When we ran the DPA analysis (see OSF) of our newly created PwNH_low-effort_, PwHL_low-effort_, and PwHL_high-effort_ groups, we replicated the results presented earlier, whereby there were no group differences in stage 1 prediction, but the PwHL_high-effort_ group was significantly slower than the PwNH group in stage 2 prediction (differing by 263 ms), and the PwHL_low-effort_ group was marginally slower than the PwNH group (differing by 104 ms). Therefore, whether we subset participants by demand (which may relate to intelligibility) or effort (in terms of subjective effort rating) the pattern remains the same. Thus it is possible than the effects of demand we report in this article could relate to induced changes in listening effort, though further study with a focused manipulation of effort is necessary to directly test this proposal.

Overall, delays in prediction with hearing loss could compound difficulty in speech-listening situations. Given that interlocutors use prediction not only in terms of what their conversation partners will say but also when they will say it (Pickering & Garrod 2007), delayed prediction for PwHL may explain some of the real-world consequences of hearing loss on conversation behavior. For example, it has been found that PwHL have slower and more variable turn-taking timing relative to PwNH (Sørensen et al. 2019; Petersen et al. 2022). If predictions of what a talker will say next are delayed, this may result in slower processing of speech (due to lack of preactivation) and thus more time being needed to comprehend an utterance and prepare a response (Corps et al. 2018). It is therefore possible that targeting and training the use of predictive processes could benefit PwHL in social situations, providing a potential route to ameliorating some of the commonly reported negative social outcomes of hearing loss (Ciorba et al. 2012). Furthermore, our findings speak to the value of hearing aid usage, since listening at a less demanding level reduced prediction delays for PwHL, suggesting not only audibility but semantic prediction benefits.

We acknowledge that while we focused on listening demand as related to hearing loss, it is also possible that increased listening demand may also delay prediction in PwNH. However, PwNH would need to be listening to speech at an unusually low level for demand to be increased to a similar degree to our PwHL_high-demand_ group. Here, our focus was on the impact of hearing loss on speech listening in everyday life; that is, how hearing loss, and potential increases in effort that may occur when PwHL listening to speech with high demand (see Ohlenforst 2017), affects speech processing. Thus, while the pure impact of listening demand is an intriguing question for further study, it was not in itself the focus of the present study. Nonetheless, it is possible that the concept of listening demand could provide a means of generalizing or comparing across somewhat disparate literatures exploring normal hearing listeners, such as complementary findings from listening to degraded speech, listening under load, and listening to a non-native language, as well as providing hypotheses for further study such as listening to quiet speech, masked speech, or foreign-accented speech.

We believe that it is important to briefly discuss how these findings may generalize more broadly to everyday language use, and the impact that the paradigm may have on behavior. The VWP involves displaying a finite set of images before hearing auditory information (in the present study 4 images are shown for 2000 ms). As a result, listeners have ample opportunity to identify and spatially encode the images on the screen before integrating the auditory information. This may limit lexical activation (to only those images on the screen) and may not draw on working memory resources in the same way as activating words without prior information, given that the critical auditory information can be mapped directly onto one of the activated images. How these findings would translate to everyday language use is not clear, but promising virtual reality research suggests that prediction occurs in more varied monologues in scenes with up to 10 referents (Heyselaar et al. 2021). We believe that extending this to more natural situations and in conversation will ensure that these findings map onto more natural language use.

In sum, this is the first study to directly test semantic prediction (in terms of preactivation of a target; see Fernandez et al. 2024b) in older adults with and without postlingual hearing loss. We individually manipulated the presentation level of spoken sentences, within a range relatively typical of conversation, to differentiate the effect of hearing loss when listening with low demand, and the effect of hearing loss when listening with high demand. We showed that while there were no differences between groups in terms of the timing of early agent-related predictions (which may rely on automatic spreading activation), there were group differences in how quickly predictions were narrowed as contextual constraint built up (which may be more strategic and resource-demanding). Although all groups made predictions, in the latter case, they were delayed with hearing loss and further by increased listening demand (with effects remaining when we instead grouped participants by effort ratings). These findings reveal both a distinction in the resource requirements of different stages of prediction and a cognitive impact of hearing loss that could be a potentially valuable area to explore for future intervention.

## ACKNOWLEDGMENTS

The authors thank Andrew McLaren for recording the stimuli.

## OPEN PRACTICES

This manuscript qualifies for an Open Data Badge. The data have been made publically available at https://osf.io/ht9yn/?view_only=624b0b040a0a48d9a45a6c1ce87ba4aa. More information about the Open Practices Badges can be found at https://journals.lww.com/ear-hearing/pages/default.aspx.

## Supplementary Material

## Footnotes

* However, see Ryskin et al. (2020) for the argument that differences in prediction across these groups stem from differing prior linguistic experience rather than differences in domain-general executive resources.

† Item examples: “Have you ever watched a game of netball” and “Is a baby kangaroo called a joey.”

‡ To ensure that any behavioral differences were not driven by this age difference, we ran early- and late-stage DPA analyses on the subset of PwNH who were 65 or older (n = 23), and all patterns remained the same. In addition, upon removing those participants the groups no longer differ in age (*p*s < 0.30), see OSF.

§ The SUBTLEX-UK database was used to establish frequency (van Heuven et al. 2014). A Zipf value of less than 3 is considered “low frequency.”

¶ Accuracy~sentence_type * group + (sentence_type|participant) + (sentence_type * group|item), family = binomial.

∥ Accuracy~sentence_type * group + (sentence_type|participant) + (sentence_type + group|item), family = binomial.

** Note that the DPA can only compute one divergence point, and the first divergence point from tree in comparison to suit would be the same as in the CP items, so we cannot explicitly analyze when preference switches from predictable target to unpredictable target. As we are interested in the increased looks to the CU target after hearing tree we must compare with a different image.

†† When looking at those participants with 100% intelligibility only, the difference between PwNH (n = 30) and PwHL_low-demand_ (n = 30 of the 32 participants) becomes marginally significant (CI: [0,200]), with PwNH diverging 102.65 earlier than PwHL_low-demand_.

‡‡ This was a study investigating early-stage prediction of a semantic target in children with prelingual hearing loss. As the stimuli were ideal for an investigation of different prediction stages, we validated the items with older UK-English speakers, with our items being adjusted slightly as a result of population-specific word choices (note the original study was run in Australia).

§§ Accuracy~group + (1|participant) + (group|item), family = binomial.

¶¶ When looking at those participants with 100% intelligibility only, the marginal difference between PwNH (n = 30) and PwHL_low-demand_ (n = 30 of the 32 participants) becomes significant (CI: [20,200]), with PwNH diverging 123.66 earlier than PwHL_low-demand_.

∥∥ Accuracy~group + (1|participant) + (group|item), family = binomial.

*** When looking at those participants with 100% intelligibility only, the difference between PwNH and PwHL_low-demand_ is no longer significant (*z* = −0.87, *p* = 0.39).
