# Supplementary material for: Effects of Hearing Loss on Semantic Prediction: Delayed Prediction for Intelligible Speech When Listening Is Demanding
Source: Ear Hear. 2025 Jun 19;46(6):1440–56. doi: 10.1097/AUD.0000000000001679 (PMC12533763; doi:10.1097/AUD.0000000000001679)
Supplement: Supplementary file 1 [file aud-46-1440-s001.pdf]

Appendix A

Sentence and image validation survey

An online survey was designed to validate the predictability for all items, and to validate the images for sub-experiment 2 (as they were previously validated on Australian rather than UK participants). Sub-experiment 1 images did not require validation as they were taken from a validated UK image database (Duñabeitia et al., 2018). The survey comprised of three tasks, which took approximately 45 minutes to complete. This included a predictability task involving picture selection for all items, a picture naming task validating the sub-experiment 2 items, and an additional task validating items that are not reported in the current study. Two counterbalanced versions of the survey were made using JISC and the items were presented in a random order, such that each participant saw half of the predictable and half of the unpredictable items (but never saw both versions of the same item). Participants were recruited using a participant database and were given a £10 Amazon voucher for their participation.

Given that the eye-tracking experiments in the current study are conducted with older L1 UK-English speakers, we validated our items with a group of participants from the same population to ensure that the items would be suitable. 31 participants took part in the primary survey (mean age 66.67 (SD=6.63); 16 male/15 female; 30 from Scotland, 1 from Northern Ireland). Two subsequent short surveys were conducted for stimuli that needed further development (detailed below), and included revised items. The first subsequent survey lasted about 30 minutes, and included 12 participants (mean age 68.00 (SD=3.88); 7 male/5 female; 11 from Scotland and 1 from Wales) who were compensated with a £10 Amazon voucher. The second subsequent survey lasted about 15 minutes, and included 15 participants (mean age 68.8 (sd = 4.73); 9 male/6 female; all from Scotland) who were compensated with a £5 Amazon voucher.

Sentence validation (Task 1)

As mentioned previously, Task 1 was a predictability task involving picture selection, validating the all items. Participants were randomly assigned to one of the two surveys. Participants were provided with the beginning of a sentence (with the critical word omitted) and instructed to choose the image (from the corresponding array of 4 images) that best completed the sentence (with each image labelled as A, B, C, or D).

The contextual constraining items with a predictable target word (CP) were highly predictable. For sub-experiment 1, the critical target image was selected 97.24% of the time (SD=4.45). For sub-experiment 2 (Holt et al.) the critical image for the CP item was selected 98.54% of the time (SD =3.27).

For the neutral filler items, 48 items corresponded to the CP items in sub-experiment 1, and 32 items corresponded to the CP items in sub-experiment 2. We set the threshold that no one item could be picked more than 60% of the time for it to be determined neutral context (since chance would be 25% of the time). We found that 24 (out of 48) of sub-experiment 1 items

and 18 (out of 32) of the sub-experiment 2 items, involved a specific image being picked 60% or more of the time. These items were then rerun in a second picture selection survey using revised stimuli. The revised stimuli were created in such a way that the critical word and visual array were the same, but the sentence frame was changed slightly to make them less predictable. For the sub-experiment 1, 22 of the 24 items now met our threshold, but 2 items failed. For the sub-experiment 2, 12 of the 18 items now met our threshold, but 6 items failed. Finally, a third survey was conducted, with the last 2 sub-experiment 1 and the last 6 sub-experiment 2 items meeting our threshold.

Therefore, for the final neutral filler items in sub-experiment 1, the target word was selected 25.05% (SD= 17.78) of the time by participants (mean collapsed over each counter-balanced image location: A: 25%, B: 25%, C: 28%, and D: 22%). For the final neutral filler items sub-experiment 2, the critical unpredictable word was selected 25.26% (SD= 16.54) of the time by participants (mean collapsed over each counter-balanced image location: A: 26%, B: 23%, C: 28%, and D: 23%).

Picture naming (Task 2)

The picture naming task was only included in the first survey and only probed the images from sub-experiment 2 (the images for sub-experiment 1 were taken from a database that was previously pre-tested with L1-speakers of UK English; see Duñabeitia et al., 2018). As one participant (of the 31 that took part in the primary survey) named only the image they provided as the answer for the sentence completion task, they were removed, leaving thirty participants' data.

For the picture naming task, the word that the participant provided for the image was coded as an exact term if it matched the word used in the Holt study, with or without additional specifying words (e.g., *fan* or *electric fan* would be coded an exact term for the image of the *fan*). 100 of the 128 images were named with the exact term by 80% or more participants (mean exact term naming = 94.99% (SD=6.54)).   The remaining 28 images were named with a related word (e.g., *tea* for *coffee*) or a semantically related word (e.g., *twig/foliage/leafy twig* for *branch*) by 80% or more of participants (mean related/semantic word naming = 96.42 % (SD = 4.45); therefore, these images were also determined to fulfilled the criteria of the category they were meant to represent.
